# PUMA screening tool to detect COPD in high-risk patients in Chinese primary care–A validation study

**DOI:** 10.1371/journal.pone.0274106

**Published:** 2022-09-09

**Authors:** Phillip Lung Wai Au-Doung, Carmen Ka Man Wong, Dicken Cheong Chun Chan, Joseph Wai Ho Chung, Samuel Yeung Shan Wong, Maria Kwan Wa Leung

**Affiliations:** 1 JC School of Public Health and Primary Care, Faculty of Medicine, The Chinese University of Hong Kong, Hong Kong, Hong Kong; 2 Department of Family Medicine, New Territories East Cluster (NTEC), Hospital Authority, Hong Kong, Hong Kong; Taichung Veterans General Hospital, TAIWAN

## Abstract

The early stage of chronic obstructive pulmonary disease (COPD) is not easily recognized. Screening tools can help to identify high-risk patients in primary care settings for spirometry and may be helpful in the early detection in COPD and management. This study aims to validate the PUMA questionnaire for use in Chinese primary care settings. This cross-sectional study recruited participants (≥40 years old, current or former smoker with ≥10 packs of cigarette per year) in primary health care clinics in Hong Kong. The Chinese version of the PUMA questionnaire was administered by trained research staff to participants awaiting consultation. COPD diagnosis was confirmed by spirometry (post-bronchodilator FEV1/FVC <0.70). A total 377 patients were recruited of which 373 completed the spirometry. The percentage of participants diagnosed with COPD (post-bronchodilator FEV1/FVC <0.70) was 27.1%. A higher PUMA score was more likely to have an advanced stage of GOLD classification (*P* = 0.013). The area under the ROC curve of the PUMA score was 0.753 (95%CI 0.698–0.807). The best cut-point according to Youden’s index for PUMA score was ≥6 with sensitivity 76.5%, specificity 63.3% and negative predictive value (NPV) 63.3%. A cut-off point of PUMA score ≥5 was selected due to higher sensitivity of 91.2%, specificity of 42.6% and high NPV of 92.7%. PUMA score performed better than CDQ and COPD-PS in the area under the ROC curve (0.753 versus 0.658 and 0.612 respectively), had higher sensitivity than COPD-PS (91.2% versus 61%) and had higher specificity than CDQ (42.6% versus 13.1%). The use of PUMA as a screening tool was feasible in Chinese primary care and can be conducted by trained staff and health professionals. The validation results showed high sensitivity and high NPV to identify high risk patient with COPD at cut-off point of ≥5. It can be useful for early detection and management of COPD.

## Introduction

Chronic obstructive pulmonary disease (COPD) is a common disease that occurs globally and affects patients’ quality of life, morbidity and mortality [1, 2]. It is characterized by chronic bronchitis and airway obstruction [3] with progressive impairment of patient’s ventilatory function [4, 5]. Patients with COPD also have a higher risk to have multiple comorbidities such as cardiovascular diseases, lung cancer which can increase mortality [6]. In addition, COPD exacerbation is a common major adverse effect that is associated with an increase in intensive care unit admission rate and mortality rate [7–9].

It had been estimated that 328 million people with COPD worldwide [10]. The overall prevalence of COPD was estimated at 5.9% in 2017 globally [11] and 6.2% in 9 Asia-pacific countries included Hong Kong [12]. In Hong Kong, COPD is the third leading cause of respiratory death after respiratory infection and cancer [13]. It accounted for 1223 deaths in 2017 [14]. Due to its health impact, early diagnosis and appropriate management is essential [15]. As the underdiagnosed rate of COPD was reported higher than 80% in 44 countries [16]. Underdiagnosed patients generally experienced fewer respiratory symptoms which can delay the diagnosis [17]. In addition, one-third of patients with COPD detected in primary care were asymptomatic or had mild symptoms only [18]. Patients may not seek medical care until the condition is severe [5, 19]. Thus, screening of high-risk patients in primary care settings is important for early detection and management of COPD. Early screening may help delay patient’s disease progression as the treatment such as tiotropium can begin at early stage that may improve the early decline in pulmonary function [20].

Screening tools can help to identify at-risk patients for spirometry and diagnosis. Several tools have been used to detect COPD in primary and secondary care and in the population e.g., COPD diagnostic questionnaire (CDQ) and COPD population screener (COPD-PS) respectively [21–24]. These can be self- administered or by health professionals. However, the accuracy e.g., sensitivity and specificity values can vary across populations [21]. The PUMA questionnaire was developed in a multicenter, multinational, cross-sectional study specifically for primary care settings in Latin America [25]. It is administered by healthcare staff. The accuracy of the PUMA cut-off point ≥5 was 76% for detecting COPD [25]. Validation results from different countries show that the cut-off point can vary [26]. There has been no prior study on the use of PUMA score to screen at-risk patients in primary care settings in Southern China and in Chinese language. The aim of this study is to determine the sensitivity and specificity of the PUMA questionnaire and cut-off points for detecting COPD in high-risk populations in Chinese primary care in Hong Kong SAR, China and compare it with other screening tools. The findings aim to validate the Chinese PUMA questionnaire and its use in Chinese primary care settings.

## Methods

### Study design

This was a cross-sectional study of eligible participants presenting to public primary care. The General Outpatient Public Clinics (GOPCs) are managed by the Hospital Authority (HA) and provide more than 80% of the primary care services to the general population in Hong Kong, including elderly and people with chronic illnesses [27, 28]. Of the New Territories East Cluster (NTEC), one of the largest clusters which provides health services to 1.3 million population (17% of the total Hong Kong population) [29], all clinics (n = 10) were invited and two clinics agreed to take part in the study. The clinics contribute approximately 34% of total primary care attendances in NTEC [30]. Ethics approval was obtained from the Joint Chinese University of Hong Kong—New Territories East Cluster Clinical Research Ethics Committee (the Joint CUHK-NTEC CREC) (CREC reference number: 2018.353). The study was in compliance with the Declaration of Helsinki. Written formal consent was obtained from all participants.

#### Inclusion criteria

Participants were considered eligible if they were a resident in Hong Kong, ≥40 years old and at risk for COPD (current or former smoker with ≥10 packs of cigarette per year) [25].

#### Exclusion criteria

Participants who had previously been diagnosed with COPD, pregnancy, contraindication for spirometry (chest, lung, abdominal or brain surgery, retinal detachment or eye surgery, hospitalization for any heart complaint in the last 3 months) or have physical or mental disabilities that render them unable to complete the study, undergoing tuberculosis treatment were excluded. The PUMA questionnaire was translated and conducted in Chinese and participants who were not able to understand Chinese were excluded.

### Study flow

Participants were approached in the waiting areas of GOPC consultation rooms and primary care respiratory clinics. The primary care respiratory clinics were located within GOPCs for early identification and intervention to the patients with COPD. Physicians will refer suspected COPD cases to the clinic to perform spirometry within three weeks. The waiting room also served general primary care physician and nurse consultations.

Participants were screened to ensure they met the inclusion criteria. Eligible participants were given a detailed explanation of the study and written consent was obtained. For participants recruited awaiting primary care respiratory clinics, spirometry was conducted on the same day. Participants recruited from the waiting area for general consultations had the option of same day spirometry or to select an appointment for spirometry at a later date. For all participants, a questionnaire was conducted by trained research staff prior to spirometry which consisted of PUMA, CDQ and COPD-PS and demographic questionnaires.

Spirometry was conducted by trained registered nurses according to the guidelines from The Hospital Authority on pulmonary function tests in GOPCs. Portable spirometers (Spirolab^®^ - MIR) were calibrated before use. Each participant completed a baseline spirometry and repeated spirometry 15 minutes later following administration of a bronchodilator (400μg salbutamol) according to the American Thoracic Society (ATS) standard [31]. The spirometry results were analyzed by the registered nurses and physicians. For participants recruited from waiting areas, they were informed by the nurse via telephone if the spirometry result was positive. A letter to a doctor was issued to the participant for further management of COPD. For participants recruited from primary care respiratory clinics, an appointment letter was issued by the clinic and were followed up according to HA guidelines.

### Recruitment response

Due to the outbreak of COVID-19 in January 2020 and the high-risk transmission during spirometry, the study recruitment ended early. Using the first 150 participants as a pilot with a prevalence 16.7%, the estimated sample size was 346 participants with precision 6%.

### COPD definition

The Global Initiative for Chronic Obstructive Lung Disease (GOLD) was used to define and classify the severity of COPD [15, 32]. Subjects who have a post-bronchodilator (post-BD) forced expiratory volume in 1s (FEV1)/forced vital capacity (FVC) < 0.70 is defined as COPD [15].

### PUMA questionnaire

The development of the PUMA questionnaire has been published previously [25]. The questionnaire consists of 7 items (S1 Table). Four items are related to objective questions of COPD risk factors: gender (ranged from 0 to 2 points), age, pack-years smoking (ranged from 0 to 2 points) and previous use of spirometry. Three items are related to subjective symptoms (each of them is ranged from 0 to 1 point): dyspnea, sputum, and cough. The highest total score is 9. Patient who has the score of ≥5 is suggested to be at risk of COPD and recommend performing spirometry [25].

The PUMA score was translated into Traditional Chinese (Cantonese) for administration, the official written and verbal language used in Hong Kong and Southern China and spoken by 88.8% of the Hong Kong population [33]. The questionnaire was translated using forward and backward translation by the research team of family physicians. The translated version was then pilot tested on 10 patients to ensure face validity included readability, consistency and face validity. All patients indicated PUMA score was clear, understandable and did not have additional comments to its format and consistency.

### Demographic questionnaire

The questionnaire included patients’ demographic characteristics, health-seeking behavior and respiratory symptoms using The Modified British Medical Research Council (mMRC) scale and COPD Assessment Test (CAT). mMRC scale consists of 5 statements to evaluate the impact of shortness of breath on activities [34]. CAT is a self-administered questionnaire used to assess the impact of COPD on daily activities and had been validated in Chinese population [35, 36]. Exacerbation history based on patient-reported in the questionnaire.

### Other screening tools

CDQ and COPD-PS were also included. CDQ is an 8-item tool with the total score range of 0–38 with a suggested cut-off at >16.5 to perform spirometry [22]. COPD-PS consists of five items on a two to five-point rating scale with the total score range of 0–10 and recommends participants to seek help from health professionals if the score is ≥5 [24]. Both CDQ and COPD-PS were translated using forward and backward translation by the research team of family physicians. The translated version was then pilot tested on 10 patients to ensure face validity included readability, consistency and face validity.

### Statistical analysis

Descriptive statistics of chi square test, independent t test were used to summarize the demographic and clinical characteristics of the participants. Spirometry results and COPD diagnostic results with GOLD classification and GOLD ABCD groups were also reported.

The validation results were reported by calculating the sensitivity, specificity, predictive positive (PPV) and negative value (PNV) at different cut-off scores. Criteria for COPD were those had a post-bronchodilator (post-BD) forced expiratory volume in 1 s (FEV1)/forced vital capacity (FVC) < 0.70. Patients with COPD were further classified by the GOLD ABCD groups, based on the mMRC scale or CAT score and the history of exacerbations in the past 12 months [15]. The optimal cut-off point was calculated by using the Youden index [37], which is the maximum value of (sensitivity + specificity -1). Receiver operator curve (ROC) was also provided to calculate the area under the curve (AUC) to determine the optimal cut-off point which optimal sensitivity [38]. The area under the ROC curve with an area higher than 0.9 has high accuracy, 0.7–0.9 has moderate accuracy, 0.5–0.7 has low accuracy and <0.5 has a chance result [39]. The internal reliability was calculated by the association of each item compared with the total score. Independent t test was used to test the differences between the mean score of the screening tools, patients with COPD and GOLD ABCD assessment tool. One way ANOVA was used to test the differences between the mean score of the screening tools and GOLD classification. Tukey’s HSD test was used as post hoc test if there were significant results from the one way ANOVA test. The statistical analysis was conducted by IBM SPSS Statistics ver 25. All *P* value ≤0.05 was considered statistically significant.

## Results

A total of 377 people participated in the study from January 2019 to January 2020, 256 (67.9%) people were recruited from the respiratory clinics in the public clinics and 121 (32.1%) people were recruited from the outpatient areas from the public clinics. Four patients (1.1%) with spirometry tests that did not meet the quality criteria. The percentage of participants diagnosed with COPD (post-bronchodilator FEV1/FVC <0.70) was 27.1%.

In general, majority of the participants were male (92.6%), were ≥60 years old (74.3%) and smoked >30 pack-year (53.6%) and performed spirometry before (56.2%). Less than half reported dyspnea (33.4%), cough (26.5%) and phlegm (36.3%). For patients with COPD, they were more likely to be ≥60 years old (*P* <0.001), smoked >30 pack-year (*P* <0.001) and have dyspnea (*P* <0.001), phlegm (*P* = 0.006), cough (*P* <0.001), had previous spirometry (*P* = 0.012), used medication to relieve respiratory symptoms (*P* <0.001), and received influenza vaccine (*P* = 0.002). Mean score of patients with COPD across the three screening questionnaires were as follows: PUMA: 6.5±1.5 versus 4.9±1.8, *P* <0.001; CDQ: 26.7±4.9 versus 23.4±6.2, *P* <0.001; COPD-PS: 5.2±1.4 versus 4.6±1.4, *P* <0.001) (Table 1).

**Table 1:** Participant demographic and clinical characteristics by patients with COPD and patients without COPD (N = 377).

	Total N = 377	(Post-BD FEV1/FVC<0.70) N = 102	(Post-BD FEV1/FVC≥0.70) N = 270	*P* value^a^
**N (%)/(mean±SD)**				
**Gender**				0.127
**Female**	28 (7.4%)	4 (3.9%)	23 (8.5%)	
**Male**	349 (92.6%)	98 (96.1%)	247 (91.5%)	
**Age**				**<0.001**
**40–49 years old**	43 (11.4%)	3 (2.9%)	40 (14.8%)	
**50–59 years old**	54 (14.3%)	6 (5.9%)	48 (17.8%)	
**≥60 years old**	280 (74.3%)	93 (91.2%)	182 (67.4%)	
**Cigarettes smoke per year**				**<0.001**
**<20 pack-year**	91 (24.1%)	12 (11.8%)	77 (28.5%)	
**20–30 pack-year**	84 (22.3%)	16 (15.7%)	68 (25.2%)	
**>30 pack-year**	202 (53.6%)	74 (72.5%)	125 (46.3%)	
**Symptoms of short of breath when walk faster**				**<0.001**
**No**	250 (66.3%)	45 (44.1%)	200 (74.3%)	
**Yes**	126 (33.4%)	57 (55.9%)	69 (25.7%)	
**Symptoms of phlegm when not suffering a cold**				**0.006**
**No**	240 (63.7%)	54 (52.9%)	184 (68.1%)	
**Yes**	137 (36.3%)	48 (47.1%)	86 (31.9%)	
**Symptoms of cough when not suffering a cold**				**<0.001**
**No**	277 (73.5%)	57 (55.9%)	218 (80.7%)	
**Yes**	100 (26.5%)	45 (44.1%)	52 (19.3%)	
**History of using spirometry**				**0.012**
**No**	212 (56.2%)	46 (45.1%)	161 (59.6%)	
**Yes**	165 (43.8%)	56 (54.9%)	109 (40.4%)	
**Dusty working environment**				0.394
**Yes**	196 (55.4%)	38 (40.9%)	120 (46.0%)	
**No**	158 (44.6%)	55 (59.1%)	141(54.0%)	
**mMRC scale**				**<0.001**
**0**	124 (34.7%)	13 (14.1%)	111 (41.9%)	
**1**	208 (58.3%)	60 (65.2%)	148 (55.8%)	
**2**	23(6.4%)	17 (18.5%)	6 (2.3%)	
**3**	2 (0.6%)	2 (2.2%)	0	
**Chronic illnesses**				
**Diabetes**				0.155
**Yes**	85 (23.7%)	17 (18.3%)	68 (25.6%)	
**No**	274 (76.3%)	76 (81.7%)	198 (74.4%)	
**Cardiac diseases** ^**b**^				0.764
**Yes**	32 (8.9%)	9 (9.7%)	23 (8.6%)	
**No**	327 (91.1%)	84 (90.3%)	243 (91.4%)	
**Hypertension**				0.106
**Yes**	188 (52.4%)	42 (45.2%)	146 (54.9%)	
**No**	171 (47.6%)	51 (54.8%)	120 (45.1%	
**Lung diseases** ^**c**^				0.449
**Yes**	8 (2.2%)	3 (3.2%)	5 (1.9%)	
**No**	351 (97.8%)	90 (96.8%)	261 (98.1%)	
**Medication history to relieve respiratory symptoms in the past 12 months**				**<0.001**
**Yes**	109 (30.2%)	48 (51.1%)	61 (22.8%)	
**No**	252 (69.8%)	46 (48.9%)	206 (77.1%)	
**Received influenza vaccine in the past 12 months**				**0.002**
**Yes**	160 (44.3%)	54 (58.7%)	106 (40%)	
**No**	201 (55.7%)	38 (41.3%)	159 (60%)	
**Use of smoking cessation products**				0.471
**Yes**	79 (21.6%)	23 (24.5%)	56 (20.9%)	
**No**	287 (78.4%)	71 (75.5%)	212 (79.1%)	
**Alcohol drinking habit in past year**				**0.010**
**Never**	190 (52.1%)	60 (63.8%)	127 (47.6%)	
**1 to 4 times per month**	117 (32.1%)	19 (20.2%)	97 (36.3%)	
**2 to 4 times per week**	58 (15.9%)	15 (16.0%)	43 (16.1%)	
**PUMA score** ^**d**^	5.2±1.9	6.5±1.5	4.9±1.8	**<0.001**
**COPD-PS score** ^**e**^	4.7±1.4	5.2±1.4	4.6±1.4	**<0.001**
**CDQ score** ^**f**^	24.3±6.0	26.7±4.9	23.4±6.2	**<0.001**

^a^Chi-squared test for categorical variables, independent t test for continuous variables.

^b^Cardiac diseases include heart failure, cardiac arrhythmia.

^c^Lung diseases include lung cancer, lung adenoma.

^d^Score of ≥5 (recommend spirometry).

^e^Score of ≥5 (recommend spirometry).

^f^Score of ≥16.5 (recommend spirometry).

Participants who were male, older, consumed more cigarettes, experienced respiratory symptoms (sputum, shortness of breath and cough) were more likely to a higher PUMA score (all *P* <0.001) (S2 Table).

Compared to COPD-PS and CDQ, a higher PUMA score was more likely with a higher GOLD spirometry grade (*P* = 0.013). Although the mean scores of COPD-PS and CDQ increased with the stage of GOLD spirometry grade, the results were not statistically significant (CDQ: *P* = 0.393, COPD-PS: *P* = 0.06). In addition, the distribution of patients with FEV1<0.70 by GOLD spirometry grade showed 93 patients (91.2%) had cut-off point of PUMA ≥5, 94 patients (96.9%) had cut-off score of CDQ ≥16.5 and 57 patients (57%) had cut-off score of COPD-PS ≥5. Although more patients with COPD were above the cut-off score, the results were not significant among all three screening tools (Table 2).

**Table 2:** GOLD classification according to airflow limitation severity in COPD.

GOLD classification^a^	GOLD 1 (n = 38)	GOLD 2 (n = 41)	GOLD 3 (n = 21)	GOLD 4 (n = 2)	*P* value^b^
	**N (%)/(mean±SD)**				
**PUMA score**	5.8±1.6	6.8±1.4	6.9±1.2	7.0±0.0	**0.013**
**CDQ score**	25.9±5.8	26.7±4.0	28.6±4.8	27.0±2.8	0.393
**COPD-PS score**	4.7±1.3	5.2±1.4	5.8±1.5	5.0±1.4	0.06
**PUMA score ≥5** ^c^	31 (81.6%)	40 (97.6%)	20 (95.2%)	2 (100%)	0.07
**PUMA score <5**	7 (18.4%)	1 (2.4%)	1 (4.8%)	0	
**CDQ score ≥16.5** ^**d**^ ^**,**^ ^**f**^	34 (94.4%)	38 (97.4%)	20 (100%)	2 (100%)	0.326
**CDQ score <16.5**	2 (5.6%)	1 (2.6%)	0	0	
**COPD-PS score ≥5** ^**e**^ ^**,**^ ^**g**^	20 (54.1%)	25 (62.5%)	11 (52.4%)	1 (50%)	0.604
**COPS-PS score <5**	17 (45.9%)	15 (37.5%)	10 (47.6%)	1 (50%)	

^a^COPD GOLD classification [32]: GOLD 1: FEV1 ≥80%; GOLD 2: 50% ≤FEV1<80%; GOLD 3: 30% ≤FEV1<50%; GOLD 4: FEV1<30%.

^b^Chi-squared test for categorical variables, one way ANOVA test for continuous variables with ≥3 independent groups.

^c^Score ≥5 (recommend spirometry).

^d^Score ≥16.5 (recommend spirometry).

^e^Score ≥5 (recommend spirometry).

^f^With missing data n = 2.

^g^With missing data n = 1.

Furthermore, by using the GOLD ABCD assessment tool, more than half (61.8%) of the participants were classified as Group A, followed by Group B (26.5%). No participant was classified as group C and 1 participant was classified as Group D. Although the mean scores of PUMA and CDQ increased with ABCD groups, the results were not statistically significant (PUMA: *P* = 0.536, CDQ: *P* = 0.711). Participants in Group B had a higher COPD-PS score (*P* <0.001). In addition, although more patients with COPD were above the cut-off points, the results were not significant among all three screening tools and ABCD groups (Table 3).

**Table 3:** GOLD groups according to ABCD assessment tool.

GOLD ABCD assessment tool^a^^,^^b^	Group A (n = 63)	Group B (n = 27)	*P* value^c^
	N (%)/(mean±SD)		
**PUMA score**	6.3±1.5	6.6±1.6	0.536
**CDQ score**	27.1±4.9	27.0±5.3	0.711
**COPD-PS score**	5.0±1.1	5.7±1.9	**<0.001**
**PUMA score ≥5** ^d^	56 (88.9%)	25 (92.6%)	0.591
**PUMA score <5**	7 (11.1%)	2 (7.4%)	
**CDQ score ≥16.5** ^e^	58 (98.3%)	25 (96.2%)	0.547
**CDQ score <16.5**	1 (1.7%)	1 (3.8%)	
**COPD-PS score ≥5** ^f^	37 (60.7%)	19 (70.4%)	0.382
**COPS-PS score <5**	24 (39.3%)	8 (29.6%)	

^a^COPD GOLD ABCD assessment tool [32]: It classifies patients with COPD to one of four groups based on exacerbation history in the past 12 months and CAT score/mMRC scale.

^b^With missing data (PUMA n = 11, CDQ n = 16, COPD-PS n = 13). Group D was excluded due to the small sample size (n = 1).

^c^Chi-squared test for categorical variables, independent t test for continuous variables.

^d^Score ≥5 (recommend spirometry).

^e^Score ≥16.5 (recommend spirometry).

^f^Score ≥5 (recommend spirometry).

### PUMA questionnaire and other screening tools to identify COPD at different cut-off points

The best cut-point according to Youden’s index for the PUMA score was ≥6. The sensitivity was 76.5% and specificity was 63.3%. However, the cut-off point of ≥5 gave greater sensitivity (91.2%) but lower specificity (42.6%). The NPV was higher with the cut-off point <5 than < 6 (92.7% versus 63.3%) (Table 4).

**Table 4:** Sensitivity, specificity, PPV, PNV for each cut-off point of the PUMA questionnaire.

	Sensitivity (%)	Specificity (%)	Youden’s Index	PPV	NPV
≥1	100	2.2	0.022	27.9	100
≥2	100	5.2	0.052	28.5	100
≥3	99	11.9	0.109	29.8	97
≥4	96.1	21.1	0.172	31.5	93.4
**≥5**	**91.2**	**42.6**	**0.338**	**37.5**	**92.7**
**≥6**	**76.5**	**63.3**	**0.398**	**44.1**	**63.3**
≥7	49	81.1	0.301	49.5	80.8
≥8	28.4	94.8	0.232	67.4	77.8
≥9	4.9	99.6	0.045	83.3	73.5

The best cut-point according to Youden’s index for the CDQ was ≥22.5. The sensitivity was 85.6% and the specificity was 43.5%. The cut-off point of ≥16.5 gave greater sensitivity (97.9%) but lower specificity (13.1%). The NPV was lower with the cut-off point <22.5 than <16.5 (89% versus 94.4%) (S3 Table). Regarding COPD-PS, the best cut-point according to Youden’s index was ≥6. The sensitivity was 34% and the specificity was 89.4%. The cut-off point of ≥5 gave greater sensitivity (61%) but lower specificity (53.3%). The NPV was lower with the cut-off point <6 than <5 (74.5% versus 78.1%) (S3 Table).

### Comparison with other screening tools

**Fig 1:**
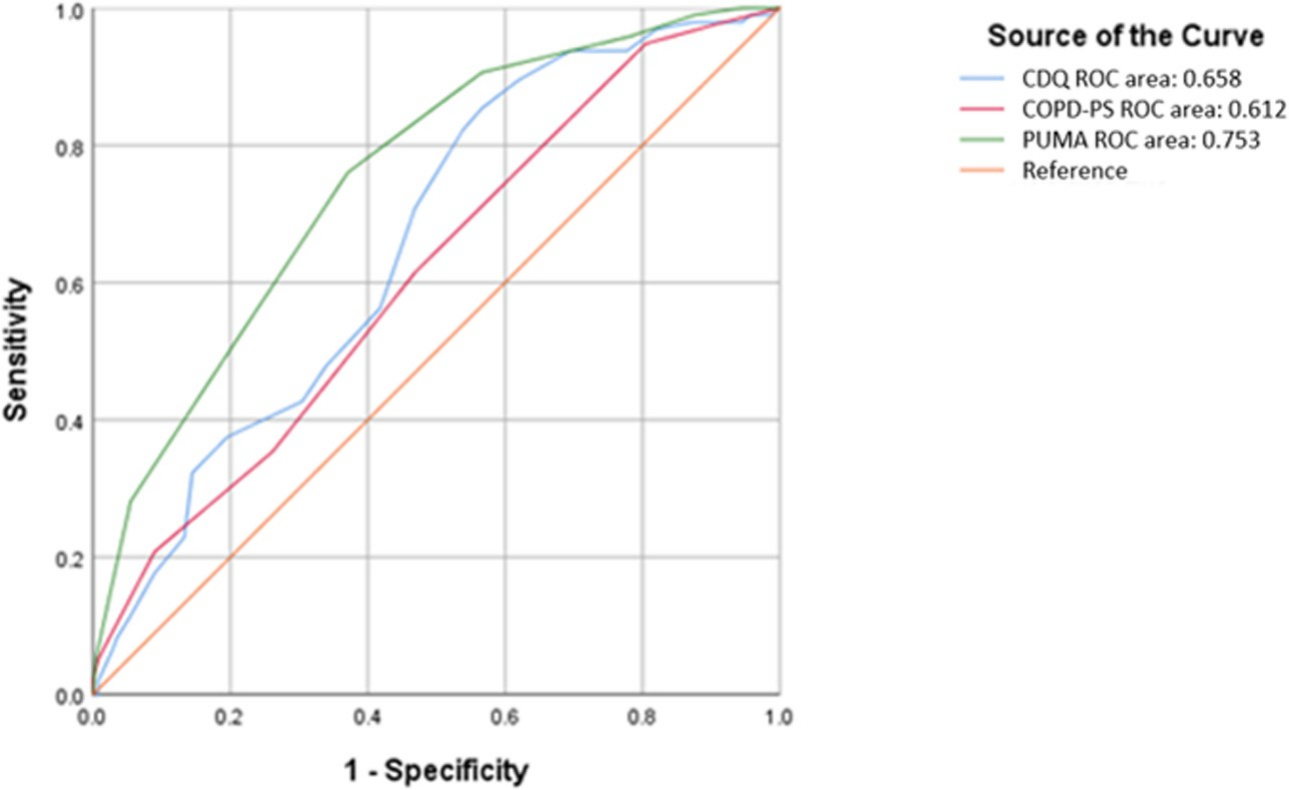
Area under the ROC for PUMA, CDQ and COPD-PS screening tools and COPD as outcome.

Using the PUMA cut-off point of ≥5, CDQ cut-off score of ≥16.5 and COPD-PS cut-off score ≥5 to compare the ROC curve, sensitivity and specificity. The ROC curve of the PUMA was classified as moderate accuracy and higher than CDQ and COPD-PS (0.753 versus 0.658 and 0.612) (Fig 1). The sensitivity of PUMA was higher than COPD-PS and slightly lower than CDQ (91.2% versus 61% and 97.9%) (S3 Table). However, the specificity of PUMA was slightly lower than COPD-PS but higher than CDQ (42.6% versus 53% and 13.1%) (S3 Table). Furthermore, PUMA had a higher PPV than CDQ and COPD-PS (37.5% versus 29.6% and 33.3%) (S3 Table).

## Discussion

This is the first validation study of the PUMA screening tool in Chinese primary care settings with other screening tools CDQ and COPD-PS. The sensitivity of PUMA in our study was higher than other studies (CDQ: 79.7%, COPD-PS: 34.9%) [22, 24]. The PPV of PUMA in our study was higher than other studies (CDQ: 18.4%, COPD-PS: 10.5%) [22, 24]. The low PPV in COPD-PS could be due to the participant recruitment from the general population instead of primary care settings [24]. Additionally, the specificity of PUMA was consistent with CDQ and lower than COPD-PS (specificity 42.6% versus 46.8% and 79.3% respectively), the area under ROC classified as low accuracy (0.756 versus 0.713 and 0.57 respectively) [22, 24]. One study validated PUMA in two different populations, one of them was a single Latin American hospital primary care center [26]. The best cut-off point of PUMA was ≥6 with sensitivity 69.9%, specificity 62.1% and PPV 59.9% [26]. When PUMA was applied to Chinese primary care setting, our results of PUMA score ≥6 showed a slightly higher sensitivity of 76.5%, similar specificity of 63.3% and a slightly lower PPV of 44.1%. Meanwhile, PUMA score ≥5 with sensitivity 85.4%, specificity 37.6% and PPV 52.9% [26]. Our results of PUMA score ≥5 showed a slightly higher sensitivity and specificity of 91.2% and 42.6% respectively and a slightly lower PPV of 37.5%. The variations may be affected by the age and smoking prevalence rate to the prevalence of COPD [24].

Our results showed that the best cut-off point of PUMA ≥6 which is higher than the original PUMA study (≥5) [25]. In the original PUMA study, 1743 current or former smokers were recruited from the primary care settings in 4 Latin American countries. Among our participants, more non-COPD participants with score 5 smoked >30 pack-years (57.1% versus 46.7%), had phlegm (23.2% versus 20%) and completed spirometry before (42.9% versus 33.3%). This may reflect the nature of patients presenting to primary care with respiratory symptoms and lower threshold to be referral to spirometry in the health care settings. Furthermore, participants recruited from the primary care respiratory clinics were more likely to be ‘referred by a doctor to have spirometry’ attributing to the higher PUMA cut-off score.

Although Youden’s index is used to determine the best cut-off point [37], an optimal cut-off point of COPD screening tool can be selected based on the combination of sensitivity, specificity, PPV and NPV results [22]. As Youden’s index may not be sensitive enough for the differences in the sensitivity and specificity, the optimal cut-off point should be selected in the context of the test [40]. For COPD screening, the detection of asymptomatic patients is important. Therefore, a high PPV and NPV is important to identify at-risk patients as much as possible to lower the risk of missing diagnosis [22, 25]. A high NPV of PUMA is considered desirable to minimise false negative results [41]. Although the best cut-point according to Youden’s index from our result was the same as the PUMA validation in a single Latin American hospital primary care center [26], our results showed that PUMA score ≥5 with a high sensitivity of 91.2%, specificity of 42.6% and high NPV of 92.7%. The PPV of cut-off point 5 from our result was similar to the original PUMA study but lower than the PUMA validation in a single Latin American hospital primary care center (PPV: 37.5% versus 34.7% and 52.9%) [25, 26]. The NPV of cut-off point 5 from our results were higher than the original PUMA study and the PUMA validation in a single Latin American hospital primary care center (NPV: 92.7 versus 90.9% and 75.8%) [25, 26]. The smaller sample size in our study (n = 377) may be accounted for the differences as the original PUMA study and the external validation study were performed on larger samples (n = 1743 and n = 974 in a single Latin American hospital primary care center) [25, 26]. In addition, it may also be attributable to the prevalence of COPD and/or smoking population, ethnic and cultural differences between Latino and Chinese populations and/or health system organisation and symptoms presentation [25, 42].

PUMA can be a useful screening tool to be administered by healthcare professionals to identify at risk patients in a short period of time as it consists of only 7 items, many of which can be integrated into history taking of the patient. It is of value in Hong Kong particularly as the average consultation time of physicians in Hong Kong public clinics was 6.7 minutes [43] and can be used in countries and situation where consultation time is limited. Other health professionals can also help to screen patients in the waiting room similar to the study logistics which can help clinics to arrange spirometry and diagnosis COPD early and initiate treatment [44].

There are several limitations to this study. Firstly, the results cannot be generalized to female smokers as they only accounted for 7.4% in this study, although the smoking prevalence of female in Hong Kong is similarly low at 4% and is much lower than western countries [45, 46]. Secondly, other screening tools has since been developed and were not included for comparison in this study e.g., CAPTURE questionnaire developed for primary care [47]. Further research can be conducted in using these tools and their effectiveness in different primary care and health care models e.g., usual referral for spirometry by physician versus routine screening by nurses/healthcare professionals and direct access to spirometry. Thirdly, the small proportion of patients with severe COPD such as GOLD 4 (n = 2), Group C (n = 0) or Group D (n = 1) may lead to type II errors and affect the statistical significance of the results. As patients with more presenting symptoms may be easily noted by physicians already and therefore not easily to encounter in our settings. Meanwhile, we were unable to conduct meaningful subgroup analysis and compare cut-off point data for the age group 40–49 years old as the numbers were too small (n = 43, 11.4% of study population), a larger study is recommended to investigate primary care screening tools, risk factors and cut-off values to enhance diagnosis in younger populations.

## Conclusions

The validation results of PUMA screening tool in Chinese primary care settings showed high sensitivity and high negative predictive value to identify high risk patient with COPD at cut-off point of ≥5. Overall, PUMA screening tool performed better than CDQ and COPD-PS in Chinese primary care in selecting at risk patients for spirometry in diagnosing COPD. The 7 item PUMA questionnaire can be used during the consultation by physicians and healthcare professionals prior to consultation or separately by healthcare professionals and clinics to directly arrange spirometry for identified at risk patients.

## Supporting information

S1 Table PUMA questionnaire. (PDF)

S2 Table Internality reliability between each PUMA items and total score. (PDF)

S3 Table The sensitivity, specificity, PPV, PNV for each cut-off point of the CDQ and COPD-PS questionnaires. (PDF)
