# Supplementary material for: PUMA screening tool to detect COPD in high-risk patients in Chinese primary care–A validation study
Source: PLoS One. 2022 Sep 9;17(9):e0274106. doi: 10.1371/journal.pone.0274106 (PMC9462562; doi:10.1371/journal.pone.0274106)
Supplement: S1 Table — (PDF) [file pone.0274106.s001.pdf]

S1 Table. PUMA questioannaire.

PUMA 評分（PUMA score questionnaire)

診所編號 (Clinic):___________

參加者編號 (Participant number):___________

項目(Items):	分數 (Score)
1. 性別 (Gender)	女 (Female)（0）<br>男 (Male)（1）
2. 年齡 (Age)	40-49 歲 (40-49 years old)（0）<br>50-59 歲 (50-59 years old)（1）<br>60 歲及以上 (≥ 60 years old)（2）
3. 吸煙狀況 (Smoking):<br>a. 您這輩子是否吸過煙? (Have you ever smoked in your life?)	沒吸多少 (No)（0）<br><20 包每年 (<20 pack-year)（0）<br>20-30 包每年 (20-30 pack-year)（1）<br>>30 包每年 (>30 pack-year)（2）
（如果受訪者一生吸煙總數少於 20 包，或在一年內每天吸煙不到 1 根，請選擇 “沒吸多少”）<br>(If the interviewee smoked less than 20 packets in a lifetime or less than 1 cigarette a day in one year, check NO)	
（包年數的算法: 吸煙年數 X 每天吸煙的根數)/20<br>(Calculation of pack-year: Years of smoking X number of cigarettes per day/20)	
平均每天吸煙的根數 (Average cigarettes per day)	吸煙年數 (Number of years smoking)
4. 當您在平地或緩坡上快步行走時，有時是否會感到呼吸急促?<br>(Do you feel short of breath at some point when you walk faster on flat ground or a small incline?)	否 (No)（0）<br>是 (Yes)（1）
5. 您是否在沒有感冒的時候也會經常從肺裡往外咳痰，或者咳痰有困難?<br>(Do you usually have phlegm coming from your lungs or difficulty expelling phlegm when not suffering a cold?)	否 (No)（0）<br>是 (Yes)（1）
6. 您是否在沒有感冒的時候一般也會咳嗽?<br>(Do you usually have a cough when not suffering from a cold?)	否 (No)（0）<br>是 (Yes)（1）
7. 是否曾有醫生或其他健康專業人士讓您往一個裝置(稱為肺量計或峰流速	否 (No)（0）<br>是 (Yes)（1）

儀)裡吹氣，以檢查您的肺功能?<br>(Have you ever been asked by a doctor or other health professional to blow into a device (called a spirometer or peak flow meter) to know your lung function?)	
**總分 (Total score)**	

≥5 分:需要進行肺量計測試 (Score ≥5: High risk of COPD and should perform spirometry)
