# Supplementary material for: PUMA screening tool to detect COPD in high-risk patients in Chinese primary care–A validation study
Source: PLoS One. 2022 Sep 9;17(9):e0274106. doi: 10.1371/journal.pone.0274106 (PMC9462562; doi:10.1371/journal.pone.0274106)
Supplement: S2 Table — (PDF) [file pone.0274106.s002.pdf]

S2 Table. Internality reliability between each PUMA items and total score.

Items	PUMA score (Mean±SD)	P value[a]
Gender		
Female	2.50±2.00	**<0.001**
Male	5.46±1.7	
Age		
40-49 years old	2.28±1.4	**<0.001[b] Post Hoc test showed all <0.001 for each groups**
50-59 years old	4.11±1.3	
≥ 60 years old	5.91±1.9	
Have you ever smoked in your life?		
<20 pack-year	3.23±1.6	**<0.001[b] Post Hoc test showed all <0.001 for each groups**
20-30 pack-year	4.73±1.5	
>30 pack-year	6.35±1.2	
4. Do you feel short of breath at some point when you walk faster on flat ground or a small incline?		
No	4.67±1.7	**<0.001**
Yes	6.37±1.7	
5. Do you usually have phlegm coming from your lungs or difficulty expelling phlegm when not suffering a cold?		
No	4.67±1.8	**<0.001**
Yes	6.23±1.7	
6. Do you usually have a cough when not suffering from a cold?		
No	4.69±1.7	**<0.001**
Yes	6.74±1.5	
7. Have you ever been asked by a doctor or other health professional to blow into a device (called a spirometer or peak flow meter) to know your lung function?		
No	4.72±1.9	**<0.001**
Yes	5.90±1.7	

[a]Independent t test for continuous variables with two independent groups

[b]One way ANOVA test for continuous variables with ≥3 independent groups, Tukey's HSD test was used as Post Hoc test
