# Supplementary material for: PUMA screening tool to detect COPD in high-risk patients in Chinese primary care–A validation study
Source: PLoS One. 2022 Sep 9;17(9):e0274106. doi: 10.1371/journal.pone.0274106 (PMC9462562; doi:10.1371/journal.pone.0274106)
Supplement: S3 Table — (PDF) [file pone.0274106.s003.pdf]

S3 Table. The sensitivity, specificity, PPV, PNV for each cut off point of the CDQ and COPD-PS questionnaires.

CDQ	Sensitivity (%)	Specificity (%)	Youden's Index	PPV	NPV
≥8.5	99	1.2	0.002	27.2	75
≥9.5	99	1.5	0.005	27.3	80
≥10.5	99	2.0	0.009	27.4	83
≥11.5	99	4.2	0.032	27.8	91.7
≥12.5	97.9	5.4	0.044	27.9	87.5
≥13.5	97.9	6.5	0.044	28.1	89.5
≥14.5	97.9	8.5	0.064	28.5	91.7
≥15.5	97.9	10.3	0.082	29.0	93.1
**≥16.5**	**97.9**	**13.1**	**0.11**	**29.6**	**94.4**
≥17.5	96.9	18.1	0.15	30.6	94
≥18.5	93.8	22.3	0.161	31.1	90.6
≥19.5	93.8	26.1	0.199	32.2	91.9
≥20.5	93.8	30.4	0.242	33.5	92.9
≥21.5	89.7	38.1	0.278	35.1	90.8
≥22.5	85.6	43.5	0.291	36.1	89
≥23.5	82.5	46.2	0.287	36.4	87.6
≥24.5	70.1	53.5	0.236	36.0	82.7
≥25.5	55.7	58.5	0.142	33.3	77.9
≥26.5	47.4	66.5	0.139	34.6	77.2

COPD-PS	Sensitivity (%)	Specificity (%)	Youden's Index	PPV	NPV
≥3	98	8	0.06	28.9	91.3
≥4	95	19.5	0.145	31.1	91.1
**≥5**	**61**	**53.3**	**0.143**	**33.3**	**78.1**
≥6	34	89.4	0.243	33.3	74.5
≥7	20	91.2	0.112	46.5	74.8
≥8	5	99.2	0.042	71.4	73.2
≥9	2	100	0.02	100	72.7
